# Enhancing AlN PMUTs’ Acoustic Responsivity within a MEMS-on-CMOS Process

**DOI:** 10.3390/s21248447

**Published:** 2021-12-17

**Authors:** Eyglis Ledesma, Ivan Zamora, Arantxa Uranga, Francesc Torres, Núria Barniol

**Affiliations:** Departament d’Enginyeria Electrònica, Universitat Autónoma de Barcelona, 08193 Bellaterra, Spain; eyglis.ledesma@uab.es (E.L.); ivan.zamora@uab.es (I.Z.); arantxa.uranga@uab.es (A.U.); francesc.torres@uab.cat (F.T.)

**Keywords:** PMUT, ultrasound, CMOS, MEMS-on-CMOS, acoustic responsivity, AlN, piezoelectric transducers, PMUT-on-CMOS

## Abstract

In this paper, guidelines for the optimization of piezoelectrical micromachined ultrasound transducers (PMUTs) monolithically integrated over a CMOS technology are developed. Higher acoustic pressure is produced by PMUTs with a thin layer of AlN piezoelectrical material and Si_3_N_4_ as a passive layer, as is studied here with finite element modeling (FEM) simulations and experimental characterization. Due to the thin layers used, parameters such as residual stress become relevant as they produce a buckled structure. It has been reported that the buckling of the membrane due to residual stress, in general, reduces the coupling factor and consequently degrades the efficiency of the acoustic pressure production. In this paper, we show that this buckling can be beneficial and that the fabricated PMUTs exhibit enhanced performance depending on the placement of the electrodes. This behavior was demonstrated experimentally and through FEM. The acoustic characterization of the fabricated PMUTs shows the enhancement of the PMUT performance as a transmitter (with 5 kPa V^−1^ surface pressure for a single PMUT) and as a receiver (12.5 V MPa^−1^) in comparison with previously reported devices using the same MEMS-on-CMOS technology as well as state-of-the-art devices.

## 1. Introduction

Currently, there is a growing demand for miniaturized devices capable of producing and sensing ultrasonic signals in a very efficient manner. There is a broad range of applications that use ultrasonics due to its non-invasive approach and small size including: minimally invasive intravascular medical imaging [1], ultrasonic powering of miniaturized implantable medical devices for in vivo and in situ physiological monitoring [2,3], systems for selective neural stimulation with an ultrasound signal [4], and fingerprints for biometric identification [5]. Some of these applications demand compact, minute systems. Piezoelectrical micromachined ultrasound transducers (PMUTs), based on out-of-plane micrometric flexural membranes are devices that can meet these requirements, thus replacing bulky piezoceramics that are difficult to compact, and result in low yield of the fabricated devices due to fabrication complexity. PMUTs composed of a multilayer laminate structure where at least one layer is made of a piezoelectric material, benefit from the robust fabrication processes used in MEMS technology, and provide high yields and scalable designs [6,7,8,9]. Moreover, PMUTs monolithically fabricated over pre-processed CMOS wafers enhance the obtention of very compact systems, are highly programmable if required and exhibit a high fabrication yield. Despite these benefits, some constraints due to the poor compatibility of PMUTs with the CMOS process, can limit the performance of ultrasonic signal processing. In addition, the CMOS process is limited to technologies that allow electrical contact from its last metal layer to the MEMS device within a specific post-process. Up until now, most of the reported PMUTs over CMOS are based on two-wafer bonding processes, in which the wafer with the PMUT (either AlN or PZT) is bonded to a CMOS wafer with analog front-end circuitry [10,11,12,13]. Despite the feasibility of this approach, the complexity of the bonding process and the limitations of the achievable fill factor impose some limitations. So far, we have already presented a system with AlN PMUTs monolithically integrated with a dedicated analog front-end circuitry for a single-pixel ultrasonic transducer [14,15]. Although we have demonstrated the viability and workability of this approach, in this paper we develop some guidelines for the optimization of the PMUTs over the CMOS and compare the results with previous ones.

The paper is divided into four sections. Section 2 presents the main parameters for the optimization of the acoustic pressure output and mechanical simulations to determine the optimal parameters of the PMUT layers. Section 3 is dedicated to the experimental electrical and acoustic characterization of the PMUTs to establish the device’s performance. In Section 4, a discussion and a comparison with the state-of-the-art PMUTs are provided.

## 2. Materials and Methods: Optimization of PMUT Transducer

PMUTs can act as acoustic transmitters and acoustic receivers. If an electrical field is applied at both sides of the membrane, a transverse stress due to the inverse piezoelectrical effect at the piezoelectrical layer is produced and bending of the membrane out-of-plane is achieved, thus producing acoustic output pressure in the media (transmitter). If an input acoustic field is applied to the membrane, it will be bent and produce a transverse stress on the piezoelectrical layer; consequently, some electrical field is produced at the sides of the membrane due to the direct piezoelectrical effect of the piezoelectrical layer (receiver). For efficient production of the acoustic or electrical signal as a transmitter or a receiver, the membrane should be excited in its first out-of-plane flexural resonant mode, which relates the dimensions, layers, and materials of the PMUT with the desired resonant frequency [16]. In this paper we focus on PMUTs working in a liquid environment in the MHz range.

To optimize the size and thickness of PMUT devices (maximum acoustic pressure output as a transducer and maximum electrical signal as a receiver), it is convenient to define a cost function or figure of merit to be optimized. For this purpose, we have defined a figure of merit (FoM) as the product of the generated output acoustic pressure, P, and generated voltage at the PMUTs electrodes, V_r_. This FoM should be maximized to enhance the PMUT’s performance. Both parameters, P and V_r_, are analyzed below.

The axial pressure amplitude at a distance z, P(z), in the far field region (z > R_0_, being R_0_, Rayleigh distance = S/λ, where S is the PMUT surface and λ is the wavelength of the acoustic signal in the propagation media, λ = c/f_0_, f_0_ = resonance frequency, c = sound speed) is given by Equation (1), where P_0_ is the pressure at the surface of the PMUT [16]. This pressure P_0_ is proportional to the membrane velocity, u_0_ = 2πd_0_f_0_ (where d_0_ is the membrane displacement) and the acoustic impedance of the media, Z_0_ = ρ_med_c (where ρ_med_ is the mass density of the acoustic media).

P(z)_far field_ = P_o_R_o_/z = u_0_Z_0_S/λ/z = u_0_f_0_ρ_med_S/z = 2πd_0_(f_0_)^2^ρ_med_S/z (1)

According to Equation (1), high resonance frequencies and large membrane displacements will benefit the acoustic pressure output of equal sized PMUTs. The membrane displacement for a PMUT is directly related to the elastic constant, k_m_, of the structure and the electro-mechanical coupling factor, η (as shown in Equation (2), where V_in_ is the actuation voltage applied between the top and bottom electrodes). Thin structures will produce larger displacement at the expense of resonating with smaller frequency because these structures will have smaller elastic constants. Analogously, when an acoustic pressure, P_a_, is applied over the PMUT membrane, the output voltage between the top and bottom electrodes of the membrane, V_r_, will be proportional to the membrane displacement, d_r_ (where r refers to receiver) according to Equation (3). In both cases it is necessary to compute the electro-mechanical coupling factor, η (Equation (4)) [16], which will quantify the conversion efficiency between the applied or received voltage and the membrane displacement. Because the PMUT is resonating, the dynamic displacement of the membrane, d = Q × d_0_ should be used, where Q is the resonator quality factor. Both displacements, static and dynamic, will be in the same range due to the high damping in the liquid environment, which reduces the Q factor to values between 1 and 3, as will be shown in the Experimental section, and it is also reported in [17].

d_0_ = ηV_in_/k_m_ (2)

d_r_ = P_a_S/k_m_, (3)

V_r_ = d_r_k_m_/η = P_a_S/η

η = 0.5e_31,f_z_p_I_piezo_ (4)

In Equation (4), e_31,f_ is the transverse effective piezoelectrical coefficient of the piezoelectrical layer, z_p_ is the distance from the mid-plane of the piezoelectric layer to the neutral axis, which depends on the multilayer lattice structure and I_piezo_ is an integral that depends on the mode shape and electrode size, which equals 5.73 for an optimized inner electrode in a square membrane [18].

The resonance frequency, f_01_, Equation (5), in a multilayered membrane depends on the resonance mode, λ_01_^2^ = 35.99 for the first out-of-plane flexural mode in square-shaped membranes [19], the membrane side, a, the flexural rigidity, D, given in Equation (6) and on the mass per unit area or surface density, µ, Equation (7). Although the membrane resonance frequency will depend linearly on the thickness, and inversely on the square of the size of the membrane, it is difficult to analyze the dependence of the resonance frequency on thickness variations in multilayered membranes with different physical properties (i.e., plate modulus, E_11_, and mass density, ρ) [16].

(5) $$f_{01}=\frac{\lambda_{01}^{2}}{2{\pi a}^{2}}\sqrt{\frac{D}{\mu }},$$

(6) $$D\approx \frac{1}{3}\cdot \overset{N}{\underset{n=1}{\sum }}E_{11,n}\cdot (\bar{h_{n}{}^{3}}-\bar{h_{n-1}{}^{3})},$$

where $\bar{h_{n}}={\;h}_{n}-Z_{\mathrm{NA}}$, is the distance from the top of the n-th layer to the neutral axis, $Z_{\mathrm{NA}}$ and h_n_ is the relative height between the bottom device and the top of the n-th layer.

(7) $$\mu_{n}=\overset{N}{\underset{n=1}{\sum }}t_{n}\cdot \rho_{n}$$

where t_n_ is the thickness of the n-th material layer.

Due to the complexity of the multilayer laminate structure for analytical computation of the above equations, finite element models (FEM) were used to evaluate the performance of the PMUT parameters of interest: displacement, d_0_; frequency, f_0_; membrane velocity u_o_ and output voltage, V_r_; and the defined figure of merit, FoM = P * V_r_.

**Figure 1:**
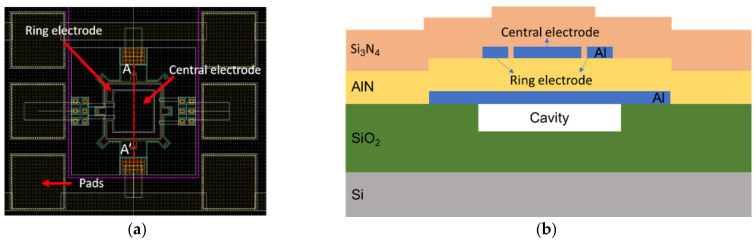
(**a**) Layout of the 80 µm × 80 µm square AlN PMUT with two top electrodes, (**b**) AA’ cross-section of AlN PMUT.

In the MEMS-on-CMOS technology considered [20,21], the PMUT is composed of four layers of different materials: AlN for the piezoelectrical layer, Si_3_N_4_ as the elastic layer, and Al for the top and bottom electrodes. Figure 1 shows the schematic cross section of the PMUT with two top electrodes: an inner or central electrode (CENT) and outer or ring electrode (RING), and one bottom electrode (BOT).

In a first approximation, an axisymmetric model of a circular PMUT was assumed and mechanic–acoustic simulations were done by means of the COMSOL finite element software. The thickness of the Si_3_N_4_ passive layer as well as the AlN piezoelectrical layer was swept from 1 to 2 µm and 0.5 to 1.5 µm, respectively, to obtain an enhanced acoustic performance in an equal-sized PMUT. The physical parameters for each of the PMUT’s layers used in the model are listed in Table 1. In the simulations, the PMUT was immersed in a liquid medium, in this case FC-70, in accordance with the experimental characterization discussed in the next sections.

**Figure 2:**
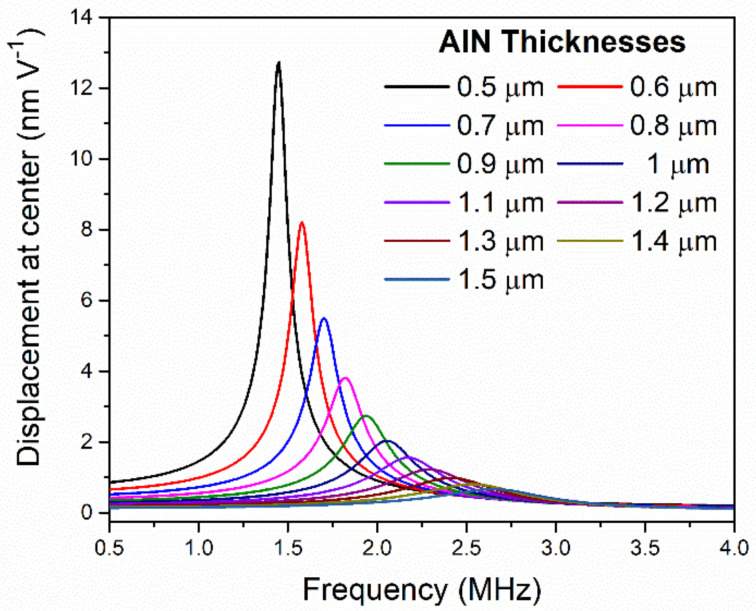
Simulated frequency response of the PMUT immersed in liquid with different AlN layer thicknesses. The normalized displacement in respect to the applied voltage is shown.

**Figure 3:**
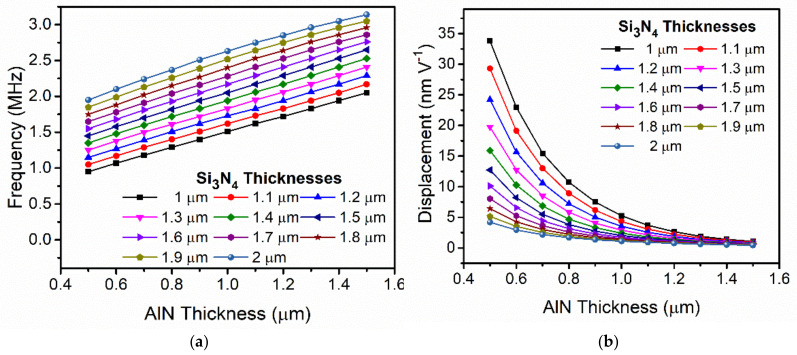
Simulated resonance frequency (**a**) and normalized dynamic displacement (**b**) of an equal-sized PMUT sweeping the AlN layer thicknesses and for different Si_3_N_4_ layer thicknesses as parameters in a liquid environment.

In Figure 2, the resonance frequency and normalized displacement as a function of the piezoelectrical layer thickness is shown (with a passive layer of Si_3_N_4_ with a thickness of 1.5 µm). As expected, thinner membranes produce larger displacements at the expense of lower resonant frequencies. In fact, resonance frequency is linearly dependent on the membrane thicknesses as is inferred from Equation (5) [22]. The dependence of resonance frequency and normalized displacement (in terms of applied voltage) as a function of the AlN and Si_3_N_4_ layer thicknesses are shown in Figure 3. This figure shows that the variation in the displacement is greater than a factor of ×77 (from 34 nm V^−1^ at minimum thicknesses to 0.44 nm V^−1^ at maximum thicknesses) while the variation in frequency does not change by more than a factor of ×3.3 (from 3.14 MHz at the maximum thicknesses to 0.95 MHz at the minimum). Accordingly, it is expected that the output pressure is maximum with the minimum thickness since the sound pressure depends on the normalized displacement and the square frequency (see Equation (1)).

**Figure 4:**
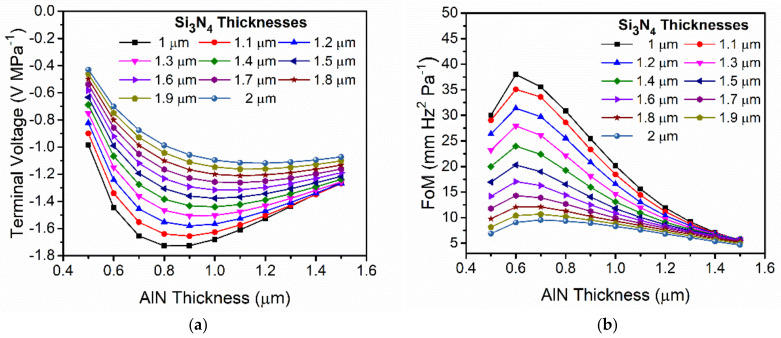
(**a**) Simulated normalized terminal voltage when an acoustic pressure is applied over the surface in a liquid environment. (**b**) Computed FoM = d_0_ × f^2^ × V_r_. Equal₋sized PMUTs were considered.

On the other hand, we are also interested in the PMUT as a receiver, thus we also simulated the normalized output voltage at the central top electrode when an acoustic pressure is applied on the PMUT surface (Figure 4). In all these simulations, the size of the top central electrode was optimized to have the maximum out-of-plane membrane displacement [18], and it was fixed for all the thicknesses. As shown in Figure 4, a maximum terminal voltage was generated at an AlN thickness close to 0.8 µm and Si_3_N_4_ thickness of 1 µm (black curve).

Once the main parameters involved in the PMUT performance as transmitter and receiver have been obtained, the figure of merit can be computed. For this computation we assumed that the acoustic pressure is proportional to d_0_ × f^2^, considering equal sized PMUTs according to Equation (1). In Figure 4b, the computed FoM = d_0_ × f^2^ × V_r_ defines an optimal point with an AlN thickness close to 0.6 µm and with a Si_3_N_4_ thickness of 1 µm.

**Figure 5:**
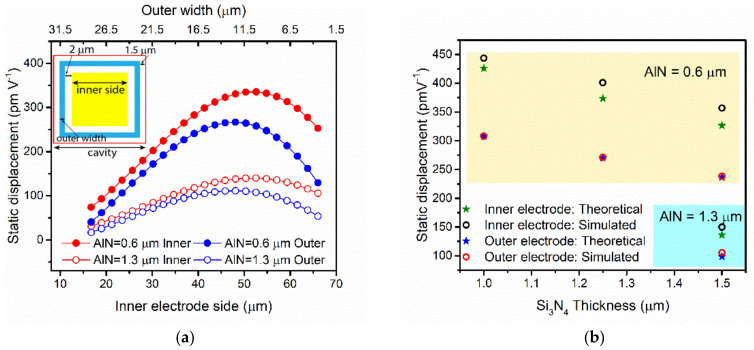
(**a**) Theoretical static displacement for two different AlN thicknesses actuating the PMUT with the inner (yellow square in the PMUT schematic top-view inset) or outer (blue ring in the inset) electrodes. Full circles indicate the 0.6 mm AlN layer, while empty circles correspond to the 1.3 mm AlN layer. (**b**) Theoretical and simulated static displacement for the proposed devices with different material layer thicknesses.

Once the optimal thicknesses for the piezoelectrical layer, AlN, and the passive layer, Si_3_N_4_ were defined, a theoretical comparison of the PMUT static behavior as a transmitter considering the two top electrodes was done. Figure 5 shows the comparison between two piezoelectrical layer thicknesses with a fixed passive layer thickness (Si_3_N_4_, 1.5 µm) in terms of normalized displacement as a function of the inner electrode side. Excitation of the membrane with the inner or the outer electrode achieves different deflections of the membrane because the outer electrode width is reduced from the optimal one, to incorporate the required gap between the inner and outer electrodes (2 µm) and the distance between the cavity and metal layer according to the technological rules for the PMUT fabrication (see schematic of the PMUT layout in the inset in Figure 5). From this static displacement, the displacement achieved with the 0.6 µm AlN was almost three times larger than with a thicker 1.3 µm AlN thickness, as expected.

Finally, to predict more realistic PMUT behaviors, 3D COMSOL simulations considering all the geometric layout and material’s layer thicknesses for the PMUT were performed. Figure 5b shows good agreement between the theoretical values computed for both electrodes: there is no difference between the theoretical and simulation results for the static displacement when the actuation is made using the outer electrodes and with a small difference with the inner electrode. From Figure 5b, it is also clear that bigger displacements are obtained with inner electrode actuation, with this difference being higher in the 0.6 µm AlN PMUT (yellow area). The main PMUT characteristics in liquid for the different proposed thicknesses are quantified in Table 2.

Several conclusions can be extracted from the results shown in Table 2. Thinner passive layers enhance the performance as a sensor but slightly decrease the output achievable pressure, mainly due to the decrease in the resonance frequency (note that the output pressure depends quadratically on the frequency according to Equation (1)). Despite the decrease in the output pressure, the FoM (considering both transmitting and receiving PMUT system) is higher for thinner PMUTs. Moreover, the performance of thicker PMUTs (i.e., 1.3 µm AlN + 1.5 µm Si_3_N_4_, was also simulated, obtaining: f = 2.48 MHz, d = 450 pm V^−1^, V_r_ = 1.34 V MPa^−1^ (normalized voltage at inner electrode), corresponding to a FoM less than 3.71 Hz^2^ m kPa^−1^), which is a factor of ×2.2 lower than that reported in the first column of Table 2, which clearly highlights the benefits of using thinner piezoelectrical layers.

## 3. Experimental Results

### 3.1. PMUTs Fabrication and Electrical Characterization

According to the optimized piezoelectrical layer thickness (0.6 µm AlN), a set of PMUTs with three Si_3_N_4_ thicknesses (1 µm, 1.25 µm and 1.5 µm) were fabricated using the MEMS-on-CMOS SilTerra technology. The results were compared with a previously fabricated PMUT with a 1.3 μm AlN piezoelectric material [18]. The MEMS-on-CMOS process from Silterra [20,21], basically consists of: (a) deposition and patterning of an stack of Al metal electrodes (top and bottom) and a physical vapor deposited AlN layer on top of the last CMOS layer; (b) releasing of the membrane through pre-defined holes around the PMUT structure; and (c) deposition of the Si_3_N_4_ elastic layer deposited with a low temperature plasma-enhanced chemical vapor deposition (PECVD) process, which allows the holes to be sealed for liquid operation.

**Figure 6:**
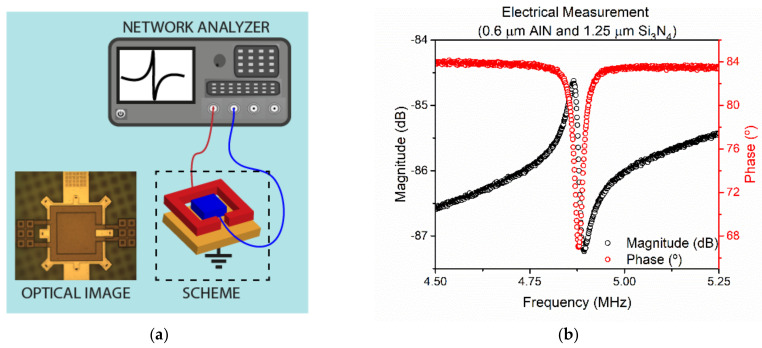
(**a**) Schematic set₋up for the electrical characterization in air and (**b**) frequency response (magnitude and phase) for the PMUT with 0.6 µm AlN and 1.25 µm Si_3_N_4_ in air.

Next, the electrical characterization in air was performed and compared with COMSOL and the analytical expression of the resonance frequency. Figure 6 corresponds to the experimental frequency response for the PMUT with 0.6 µm AlN and 1.25 µm Si_3_N_4_ thicknessess, an optical image is shown as an inset in the characterization set-up (Figure 6a).

From Figure 6b, an effective electromechanical coupling factor, k_eff_^2^ = 1.14% was computed according to Equation (8) [23,24], between the inner and outer electrodes where f_s_ = 4.866 MHz (resonance) and f_p_ = 4.894 MHz (antiresonance or parallel resonance). In Table 3, the resonance frequencies for the three squared PMUTs are shown together with those computed by Equation (5), and the FEM simulated ones, and show a good match.

k_eff_^2^ = (f_p_^2^ − f_s_^2^)/f_p_^2^ (8)

### 3.2. Acoustic Characterization

The PMUTs were characterized as an acoustic transmitter and receiver in a liquid environment (FC-70, sound speed c = 689 m s^−1^ and mass density, ρ = 1940 kg m^−3^). As transmitters, the PMUTs were driven by four cycles of a 22V_pp_ harmonic signal generated by the signal generator (Keysight 81150A, Sunnyvale, CA, USA). Both top electrodes (inner and outer) were independently polarized for a complete characterization with the bottom electrode grounded. The acoustic pressure was measured with a commercial hydrophone from ONDA, Sunnyvale, CA, USA (HNC-1500) and displayed on an oscilloscope (Keysight DSOX3054A, Sunnyvale, CA, USA). Frequency and hydrophone micrometric positioning over the PMUT were manually tuned to maximize the receiving signal. Note that for liquid operation, the added mass loading effect should be considered [25], which will lower the resonance frequency from the 4–5 MHz found in air to the 1–2 MHz range as expected from the results shown in Figure 3.

**Figure 7:**
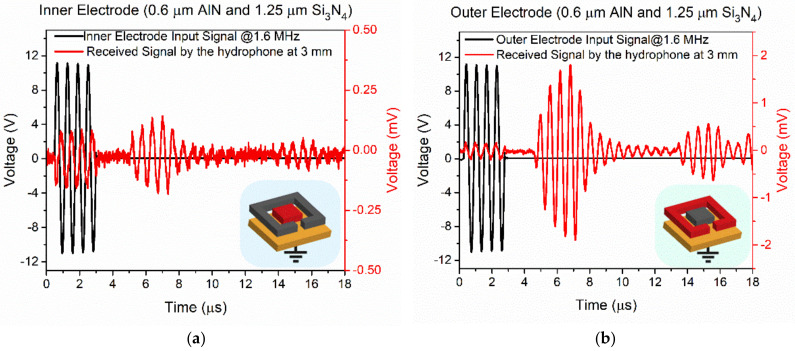
Time response of the acoustic signal produced by the 0.6 µm AlN and 1.25 µm Si_3_N_4_ thicknesses PMUT driving (**a**) the inner electrode, and (**b**) the outer electrode (in both cases, the bottom electrode is grounded). Left axis is the applied voltage to the PMUT (black), right axis is the voltage received by the hydrophone (red). Two echoes were taken with the hydrophone situated at 3 mm over the PMUT surface.

**Figure 8:**
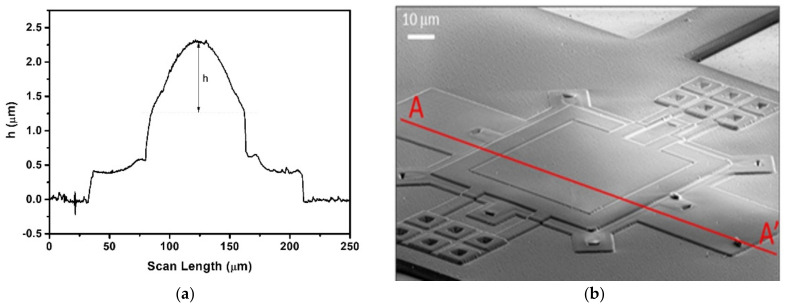
(**a**) Profile for curvature characterization of the 0.6 µm AlN and 1.25 µm Si_3_N_4_ PMUT over the red line in the (**b**) SEM image, showing a h = 1 µm height in the middle of the membrane.

**Figure 9:**
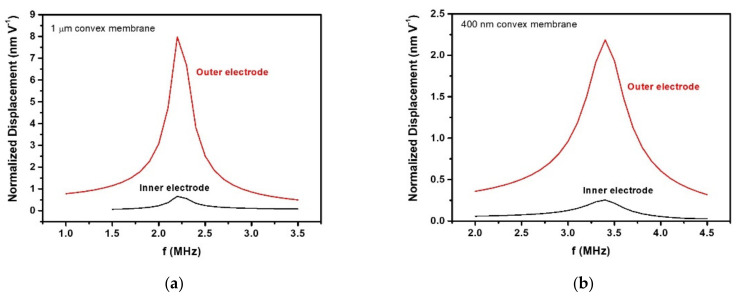
FEM simulations to compare displacement as a function of the driving electrode (inner or outer) when a convex membrane with a maximum height at the center equal to 1 µm (**a**) and 400 nm (**b**) was considered.

The signals received by the hydrophone exciting the PMUT with 0.6 µm AlN and 1.25 µm Si_3_N_4_ thicknessess at 1.6 MHz are shown in Figure 7. Unexpectedly, driving the inner electrode (Figure 7a) produces lower amplitude signals than driving the outer electrode (Figure 7b). FEM simulations from the previous section predicted the contrary as the size of the electrodes are complementary and were chosen to maximize the movement. This behavior can be attributed to the additional curvature produced on the PMUT surface by the residual stress from the piezoelectric, passive layer and electrodes during the fabrication. The physical characterization of the surface profiles of the PMUTs using a surface profilometer confirmed that thinner membranes are more prone to bending (Figure 8) [26,27]. The maximum central height for the 0.6 µm AlN and 1.25 µm Si_3_N_4_ thicknessess was 1 µm (see Figure 8) while the same feature decreased to 200 nm in the case of 1.3 µm AlN and 1.5 µm Si_3_N_4_. Several FEM simulations were done with same-sized membranes with two top electrodes and two different curvatures (maximum central heights of 1 µm and 400 nm). Figure 9 shows the simulated displacements, which are higher when the outer electrode is driven in both cases and with higher movement when the central height is bigger, which is in line with the obtained voltage amplitudes shown in Figure 7.

Table 4 summarizes the performance of the fabricated PMUTs. The output pressure was measured at 2 mm using the HNC-0200 ONDA hydrophone considering its sensitivity at the operation frequency and under the same voltage driving conditions (four cycles and 22 Vpp). From the normalized acoustic pressure in respect to applied voltage, the normalized surface pressure (P_0_ = P × distance/R_0_, where R_0_ is the Rayleigh distance already defined) can be calculated. This normalized surface pressure is an important parameter for the assessment of the performance of the presented PMUTs as transmitters and allows the comparison with the state-of-the-art PMUTs, as will be discussed in Section 4. As expected, thinner PMUTs with 0.6 µm AlN result in higher surface pressure, being almost a factor of 2 in comparison with the thicknesses used in previous works using the same technology. In addition, the influence on the achieved surface pressure is weakly related to the Si_3_N_4_ layer thicknesses.

**Figure 10:**
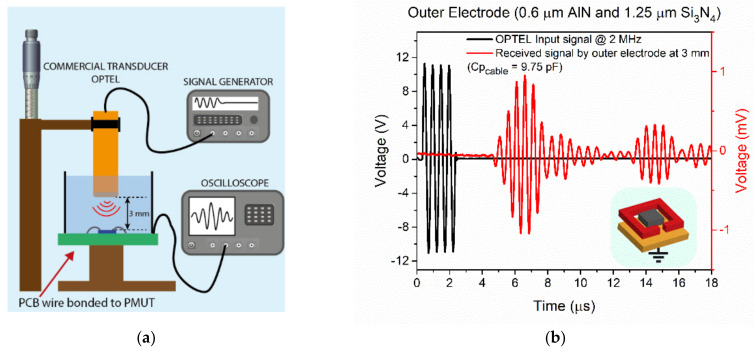
(**a**) Schematic set₋up for the acoustic characterization as sensor and (**b**) characterization of the transient signal received by the 0.6 µm AlN and 1.25 µm Si_3_N_4_ PMUT acting as an acoustic sensor when a commercial ultrasound transducer is used.

The PMUTs devices were also characterized as sensors using a commercial ultrasound transducer (OPTEL) at a 3 mm distance in the liquid environment. The transducer was excited with four cycles at the central resonance frequency of each of the PMUTs, with four cycles and 22 V_pp_. The generated signal in the outer electrode directly acquired by the oscilloscope is shown in Figure 10 for the case of a 0.6 µm AlN and 1.25 µm Si_3_N_4_ PMUT. The same measurements under the same conditions were done for all the PMUTs to complete the comparison (Table 4, Received voltage column). The amplitudes decrease for thicker piezoelectric layers, with a weak dependence in respect to the thickness of the passive layer as was already seen in the simulations. Finally, the FoM was computed in this table, and demonstrated better performance for the thin PMUTs as was already predicted by the FEM simulations.

## 4. Discussion and Conclusions

As was shown in the previous sections, the presented PMUTs with reduced thicknesses for the piezoelectrical and elastic layer, achieve better performance as transmitters as well as receivers in comparison to PMUTs of equal size with the same technology but larger thicknesses. In this section, we compare the PMUTs’ performance with several recently published papers on PMUTs. For convenience, the receiving sensitivity will be used. The receiving sensitivity is computed considering the calibration of the used ultrasound transducer at distances of 3 mm and 2 MHz [18].

A summary is given in Table 5. In comparing the same technological process, which follows a MEMS-on-CMOS process and exactly the same PMUT layout [18], the presented PMUTs exhibit bigger transmission and receiving sensitivities, with an overall x4 enhancement factor. This enhancement was also demonstrated in comparison with other PMUTs based on AlN, either with CMOS compatible processes [28] for the fabrication of PMUTs together with CMOS circuitry or with non-CMOS compatible processes [5] in which complex bonding between PMUT devices and CMOS circuitry are required. On the other hand, the presented PMUTs offer lesser performance in comparison with PZT devices [29] as transmitters, due to the high piezoelectrical constant of this material [6]. Despite this, the FoM is only a factor of ×1.8 smaller, which can be overcome by considering the compactness of the system due to the monolithic integration with the CMOS circuitry in our process. Note also that only simulated results are given for the receiver sensitivity in the case in [29]. In summary, the thinner PMUT with 0.6 µm AlN thickness and 1 µm Si_3_N_4_ thicknesses presented in this work, demonstrates higher acoustic pressure production along with higher receiver sensitivity, and enhances the applicability of PMUTs on CMOS for ultrasound systems that need to be miniaturized.

## Figures and Tables

**Table 1:** Material properties and thicknesses used in COMSOL-FEM simulations.

PMUT Layer	Properties			Geometric Dimensions	
	Mat.	Young’s Modulus (GPa)	Density (kg m^−3^)	Side (µm)	Thickness (µm)
Substrate	SiO_2_	70	2200	100	2
Bottom Electrode (BOT)	Al	70	2700	86	0.4
Piezoelectric	AlN ^1^	279	3230	100	0.5 to 1.5
Top Electrode (CENT)	Al	70	2700	56.6	0.35
Top Electrode (RING)				External side: 77 Internal side: 60.6	0.4
Passive	Si_3_N_4_	250	3100	100	1 to 2

^1^ The piezoelectric coefficients e_33_ and e_31_ used in COMSOL are 1.55 C m^−2^ and −0.6 C m^−2^, respectively.

**Table 2:** Simulated performance for different Si_3_N_4_ layer thicknesses for a PMUT with 0.6 µm AlN piezoelectrical layer using the inner or outer electrodes in liquid.

Si_3_N_4_ Thickness	1 µm		1.25 µm		1.5 µm	
Electrode	Inner	Outer	Inner	Outer	Inner	Outer
Frequency, f (MHz)	1.36		1.58		1.8	
Displacement, d (pm V^−1^)	2309	1539	1809	1175	1431	914
d × f^2^ (m V^−1^ Hz^2^)	4271	2846	4516	2933	4636	2961
Terminal voltage ^a^, Vr (V MPa^−1^)	1.91	1.78	1.73	1.55	1.54	1.35
FoM (Hz^2^ m kPa^−1^)	8.15	5.06	7.81	4.55	7.14	3.99

^a^ Normalized value when 1 Pa is applied over PMUT surface.

**Table 3:** Resonance frequencies in air for the PMUTs with different layer thicknesses.

Layer Thickness		Resonance Frequency (MHz)		
AlN (µm)	Si_3_N_4_ (µm)	Experimental	COMSOL	Analytical
0.6	1	4.47	4.19	4.18
0.6	1.25	4.87	4.67	4.69
0.6	1.5	5.21	5.14	5.20

**Table 4:** Experimental performance characterization of the PMUT as an ultrasound transmitter and receiver using the outer electrode for electrical actuation/sensing. The last column shows computed FoM.

Layer Thickness		Frequency (MHz)	Normalized Pressure @ 2 mm (Pa V^−1^)	P_0_, Normalized Surface Pressure (kPa V^−1^)	V_r_, Received Voltage (mV_pp_)	FoM P_0_ V_r_ (Pa)
AlN (µm)	Si_3_N_4_ (µm)					
0.6	1	1.5	33.8	4.9	2.8	13.7
0.6	1.25	1.6	36.2	4.8	2.5	12
0.6	1.5	2	33.4	3.6	2	7.2
1.3	1.5	2.4	27.4 *	2.4	<1	<2.4

* PMUT driving voltage in this case was a 32 Vpp squared signal instead of 22 V_pp_ sine signal.

**Table 5:** Comparison of PMUT performance in liquid operation.

Parameters	[5] 2017	[28] 2018	[29] 2018	[18] 2020	This Work
Transducer	AlN 30 µm × 43 µm	AlN 50 µm × 50 µm	PZT 80 µm × 80 µm	AlN ^a^ 80 µm × 80 µm	AlN 80 µm × 80 µm
Array/single	Array 1 × 56	Array 3 × 20	Single	Single	Single
Process	Bonded-to-CMOS	CMOS compatible	Bonded-to-CMOS	MEMS-on-CMOS	MEMS-on-CMOS
ST (kPa V^−1^)	2.95	2.93	27 ^b^	1.9	4.9
SR (V MPa^−1^)	2 ^c^	510 ^d^	4 ^e^	7.6	12.5
FoM (×10^3^)	5.9		108	14.4	61

^a^ Clampled-clamped square PMUT (same layout as presented here). ^b^ Estimated according to the data provided in [29] and considering ST = P × distance/R_0_ = 27 kPa V^−1^ if experimental data are used (extracted from [29]: Pressure = 8 kPa/30 V = 0.3 kPa V^−1^, distance = 5 mm, and R_0_ = (80 µm)^2^/λ = 55.6 µm where λ = c/f = 1500 m s^−1^/13 MHz = 115 µm). ^c^ Value obtained together with the custom CMOS ASIC [5]. ^d^ In reception 10 V pC^−1^ charge amplifier was used [28]. ^e^ PZFlex simulations [29].

## Data Availability

The data presented in this study are available on request from the corresponding author.
